# Stable Isotopes and Zooarchaeology at Teotihuacan, Mexico Reveal Earliest Evidence of Wild Carnivore Management in Mesoamerica

**DOI:** 10.1371/journal.pone.0135635

**Published:** 2015-09-02

**Authors:** Nawa Sugiyama, Andrew D. Somerville, Margaret J. Schoeninger

**Affiliations:** 1 Department of Anthropology, National Museum of Natural History, Smithsonian Institution, Washington, District of Columbia, United States of America; 2 Department of Anthropology, University of California, San Diego, La Jolla, California, United States of America; University College Dublin, IRELAND

## Abstract

From Roman gladiatorial combat to Egyptian animal mummies, the capture and manipulation of carnivores was instrumental in helping to shape social hierarchies throughout the ancient world. This paper investigates the historical inflection point when humans began to control animals not only as alimental resources but as ritual symbols and social actors in the New World. At Teotihuacan (A.D. 1–550), one of the largest pre-Hispanic cities, animal remains were integral components of ritual caches expressing state ideology and militarism during the construction of the Moon and the Sun Pyramids. The caches contain the remains of nearly 200 carnivorous animals, human sacrificial victims and other symbolic artifacts. This paper argues the presence of skeletal pathologies of infectious disease and injuries manifest on the carnivore remains show direct evidence of captivity. Stable isotope analysis (δ^13^C and δ^15^N) of bones and teeth confirms that some of these carnivores were consuming high levels of C_4_ foods, likely reflecting a maize-based anthropocentric food chain. These results push back the antiquity of keeping captive carnivores for ritualistic purposes nearly 1000 years before the Spanish conquistadors described Moctezuma’s zoo at the Aztec capital. Mirroring these documents the results indicate a select group of carnivores at Teotihuacan may have been fed maize-eating omnivores, such as dogs and humans. Unlike historical records, the present study provides the earliest and direct archaeological evidence for this practice in Mesoamerica. It also represents the first systematic isotopic exploration of a population of archaeological eagles (n = 24) and felids (n = 29).

## Introduction

Spanish conquistadors in the sixteenth century left behind detailed accounts of the extensive breeding facilities managed for the Aztec ruler, Moctezuma [1, 2]. In his *Segunda Carta de Relación*, Hernan Cortés describes two structures dedicated to the import and breeding of diverse carnivorous beasts (wolves, pumas, jaguars, lynxes, foxes, rattlesnakes, etc.) and birds (raptors and other tropical, freshwater, and marine birds) [3]. By this time the Aztecs were successfully capturing and even breeding wild animals in the capital, which hosted three hundred specialists dedicated to their care. This captive population probably fueled the rich offering caches at the ceremonial center in the Templo Mayor (Great Temple), where these very same species were excavated [2, 4]. This paper examines another grand ceremonial center preceding the rise of the Aztec empire to understand when such specialized animal management programs developed in Mesoamerica and to reconstruct what this initial experimentation period would have looked like.

Teotihuacan (A.D. 1–550), one of the largest ancient urban centers in the New World, is located 45 km northeast of modern day Mexico City. This World Heritage site is known for its centralized city-grid layout [5, 6], particularly in the ceremonial precinct where three major pyramids testify to Teotihuacan’s role as an important political and ritual center. Recent tunnel excavations inside the Moon Pyramid [7, 8] and the Sun Pyramid [9, 10] encountered a series of dedicatory offerings that were put in place at various stages of their building sequences. The contents of these offerings were premier expressions of state ideology and militarism, containing symbolically powerful artifacts such as carved obsidian, shell and greenstone objects, human sacrificial victims, and the remains of many carnivores [8, 11].

Animals were an integral and abundant component of these ritual caches at Teotihuacan, occurring in quantities often in accord with cosmologically significant numbers related to the Mesoamerican calendric cycle. With at least 194 animals (Minimum Number of Individuals, MNI) deposited in the offerings at the Moon Pyramid and Sun Pyramid, this assemblage attests to the important role animals played in state-level ritualized activities. Furthermore it provides one of the most prominent examples of mass animal sacrifice in Mesoamerica and is only comparable to the aforementioned Late Post-Classic caches at the Aztec ceremonial center of Tenochtitlan (A.D. 1325–1521) [4, 12].

For the Teotihuacan project we reconstructed complex life-histories for the animals including means of acquisition, management, preparation, use and discard. For this, we recorded body positions, element representations, surface modifications, and performed stable isotope analyses. Both zooarchaeological and isotopic data confirm the presence of a heterogeneous population among the carnivores utilized in state rituals; some were kept in captivity for prolong periods of time, while others were pulled from a wild population. This transformation in human-predator dynamics documents a critical moment when humans began to control animals not just as alimentary sources, but also as highly symbolic beasts for specifically ritual purposes.

## Materials and Methods

Four dedicatory caches from the Moon Pyramid (assigned Entierros 2, 3, 5 and 6) and one in the Sun Pyramid (Ofrenda 2) were studied. The former was explored as part of a seven year extensive excavation program by the Moon Pyramid Project directed by Saburo Sugiyama and Ruben Cabrera [7, 13]. Five burial/offering complexes, designated Entierros 2 through 6, were assigned to one of the seven construction episodes defined by the project [7, 8]. Of these, four caches contained animal remains.

The latter monument was explored as part of the Program of Investigation and Consolidation at the Architectural Complex of the Sun Pyramid, Teotihuacan (Sun Pyramid Project) from 2005 to present by Alejandro Sarabia [9, 10]. Each offering was built in dedication to a new building phase; at the Moon Pyramid these caches were associated with Buildings 4 to 6, and the Sun Pyramid Ofrenda 2 was placed as a foundation to the construction of the main corpus of the monument (Table 1).

**Table 1:** Dedicatory contexts described in the text and their associated dates.

Dedicatory cache	Building phase	Date
*The Moon Pyramid*		
Entierro 2, 6	Building 4	A.D. 250±50
Entierro 3	Building 5	A.D. 300±50
Entierro 5	Building 6	A.D. 350 ± 50
*The Sun Pyramid*		
Ofrenda 2	Sun Pyramid	A.D. 150–300

A full zooarchaeological study of the collection was completed at the Paleozoology laboratory at the National Autonomous University of Mexico (UNAM) under the guidance of Raúl Valadez and his team. These materials were analyzed with the approval of the project directors of the Moon Pyramid Project (Saburo Sugiyama and Ruben Cabrera) and Sun Pyramid Project (Alejandro G. Sarabia). The zooarchaeological assemblage from the Moon Pyramid and Sun Pyramid dedicatory caches are currently housed at Arizona State University’s Teotihuacan Research Laboratory in San Juan Teotihuacan, Mexico. Species identification, MNI calculation, skeletal element representation and especially surface modifications (pathologies and cutmarks) were recorded following standard zooarchaeological practice [14].

Upon completing the zooarchaeological investigation, stable isotope analysis was implemented to further distinguish wild versus captive populations through paleodietary reconstructions. Carbon stable isotope ratios reflect different plant photosynthetic pathways (C_3_, C_4_ and CAM), with higher δ^13^C_collagen_ and δ^13^C_apatite_ values indicating a larger contribution of C_4_ or CAM plants to the diet [15, 16]. While there are several C4 and CAM plants present in arid highland environments of Mexico [17], the main C_4_ plant exploited in this region was agricultural maize (*Zea mays*). Nitrogen stable isotope ratios are strongly influenced by the tropic position of the organism [18, 19], and generally speaking, elevated δ^15^N_collagen_ values suggest a higher trophic position. Thus, monitoring carbon and nitrogen isotopic composition can illuminate degrees of human intervention in the animal’s diet. In the New World, animals raised in captivity, like macaws penned at the pre-Hispanic site of Paquimé in northern Mexico [20] or turkeys in the ancient Southwestern United States [21], were identified isotopically because of their unnaturally high reliance on C_4_ based resources. At the ancient Maya site of Colha, isotopic analysis of dog bones showed that increased reliance on C_4_ based resources correlated with a decrease in nitrogen values, suggesting lower levels of carnivory [22].

Destructive analysis for the isotopic study was authorized by the INAH (National Institute of Anthropology and History, Mexico), document number 401-3-10136. Most of the samples were destroyed at the time of isotopic analysis or altered into pure collagen or apatite. The left-over fragments continue to be housed at the Paleodiet laboratory, Anthropology Department in the University of California, San Diego. See a full list of specimen numbers and the isotopic results in the Supporting Online Materials (S1 Table).

Only bones of eagles, canids (wolves), felids (jaguars and pumas) and leporidae (rabbits and hares) were included for the isotopic study. When feasible, all species were sampled from each context including both primary (whole) and secondary (prepared faunal artifacts) deposits. In addition samples that capture changes in dietary patterns between early (tooth) and later (bone) periods of the same organism were taken on 43 individuals. The total number of individuals included in the study (n = 83) and the quantity of bones and teeth selected (n = 142) provide a representative sample across both species and burial contexts.

Strict criteria to document diagenesis for both collagen (% collagen yield greater than 1% and C/N ratios between 2.9 and 3.6) [23] and apatite (C/P ratios above 0.1 and IR-SF below 4.0 for bone apatite) [24–26] were set following pertinent literature (S2 Text). Fifty bone apatite, 55 tooth apatite, and 51 bone collagen results are included in the study (S2 Table). Unfortunately this resulted in no bone (apatite and collagen) samples from Entierro 5 and only two bone apatite and one bone collagen samples from Entierro 3. Tooth enamel apatite provides some of the only specimens from these two contexts.

Isotope samples were processed at the Paleodiet Laboratory in the Anthropology Department at the University of California, San Diego and analyzed at the Scripps Institute of Oceanography (SIO) Analytical Facility. Interior and exterior surface and all spongy bone was cleaned with a diamond point Dremel bit or stainless steel engraving bit. Ultrasonic baths in double distilled H_2_O (two times) and acetone proceeded this initial surface scraping, at which point most consolidants and glues were no longer visible. If consolidants were still apparent, extra washes in double distilled H_2_O and acetone were added. In the case of teeth, ultrasonic baths were carried out first to loosen all surface consolidants on the enamel, and once dried a section of the enamel interior and exterior surface was ablated with the drill. For teeth samples, only enamel apatite was analyzed for the present study. Based on research assessing the effects of B-72 [27] and our own experimental project on Mexican consolidants (Reconos^110^ and Reconos^220^) and conventional Mexican glue (Resistol 850) applied to this collection, it was determined that surface cleaning methods applied were sufficient to rid the samples of their respective consolidants.

Mineral apatite samples were processed using procedures similar to Koch et al. [28]. A small fragment of cleaned and dried bone or tooth was removed and ground into a fine powder using an agate mortar and pestle, and 35mg of this powder was put into micro-centrifuge tubes. All organics were then dissolved by soaking powder in 2% sodium hypochlorite (bleach) (NaClO) solution for 24 hours. Samples were rinsed three times with double-distilled/deionized water, and were then reacted with 0.1M acetic acid (C_2_H_4_O_2_) for 24 hours. These were then rinsed with double-distilled/deionized water three times and dried at 50°C for 24 hours [24]. Two mg of powder were transferred to glass tubes, reacted with phosphoric acid (H_3_PO_4_), and analyzed on a Gas Bench Thermo MAT 253 connected to a Thermo-Finnigan Delta XP Plus mass spectrometer. An internal CaCO_3_ standard calibrated to NBS-18 and NBS-19 was run over the course of six months resulting in a reproducibility of ±0.2σ for δ^13^C. δ^13^C_apatite_ results are presented relative to the Pee Dee Belemnite (PDB) international standard.

Collagen samples were prepared following protocols described by Schoeninger et al. [29]. Only bone samples underwent collagen extraction, whereby cleaned bones were demineralized with 0.25M hydrochloric acid (HCl) over a course of several weeks at room temperature. The HCl was changed every two days. Upon demineralization, samples were neutralized through five rinses of double-distilled/deionized water. Humic acids were removed through soaking the collagen in 0.125M sodium hydroxide (NaOH) for 24 hours. Subsequently samples were rinsed an additional five times with double distilled/deionized water. Collagen was then solubilized by placing samples in pH3 HCl, heating them to 75°C, and transferring the liquid into Teflon beakers to dry over the course of several days. Samples were then resolibilized with pH3 HCl, frozen, and lyophilized in scintillation vials. Once weighed, collagen samples were analyzed with a Conflow and Costech 4010 Elemental Analyzer coupled to a Thermo-Finnigan Delta XP Plus Mass Spectrometer at the SIO stable isotope analytical facility through a double continuous-flow (CF) IRMS combustion. Peedee belemnite (PDB) marine limestone standard for carbon and atmospheric N_2_ (AIR) standard for nitrogen were utilized with a reproducibility of ±0.2σ for δ^13^C and δ^15^N.

A couple of data corrections compensate for material offsets as well as to account for the few modern comparative samples included in the study. Warriner and Tuross [30] have demonstrated that enamel apatite tends to be enriched over bone by 2.3‰ in carbon and 1.7‰ in oxygen values. Thus tooth isotopic ratios were corrected to match bone samples in all of the figures presented in the study (note S1 Table presents raw data prior to data corrections). When comparing modern skeletal isotopic ratios to archaeological ones, we must account for depletion in atmospheric ^13^CO_2_ in modern versus pre-industrial periods as a result of burning fossil fuels [31]. Thus all modern δ^13^C bone values were adjusted positively by 1.5‰ in the text and figures.

## Results and Discussion

### Zooarchaeology

**Fig 1:**
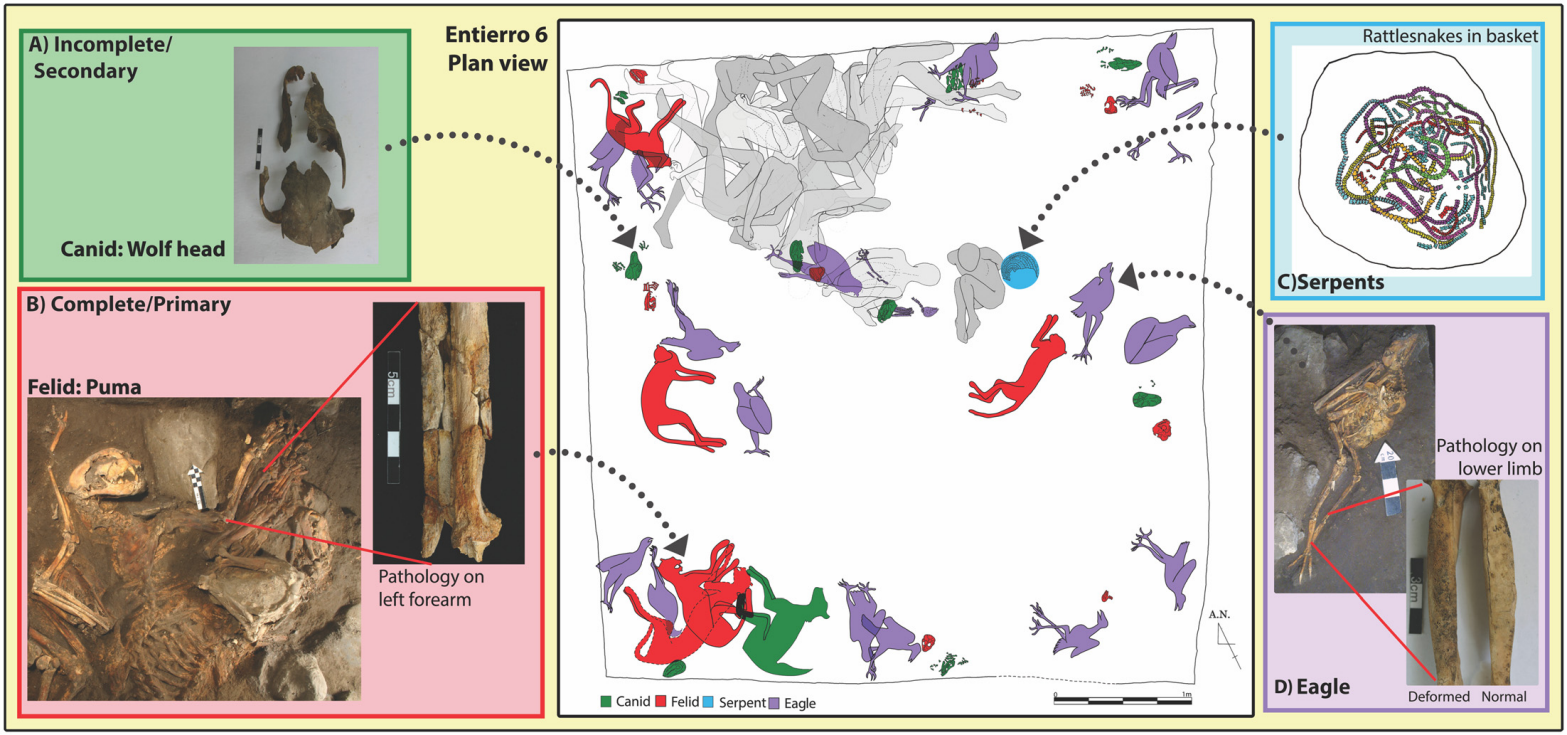
Plan view reconstruction of the animal (color) and human (grey) remains from Entierro 6, Moon Pyramid (center). (Human reconstruction drawn by G. Pereira, animals drawn by N. Sugiyama). A) Incomplete canid cranium, B) complete felid with a pathology on left forearm (fused radius and ulna), C) plan view drawing of entwined rattlesnake remains inside a basket (drawing by N. Sugiyama), D) eagle skeleton with pathology on left lower limb (tarsometatarsus).

A total of 66 complete sacrificed animals and 127 bone artifacts (incomplete animal parts) were distributed throughout the five dedicatory caches analyzed (Table 2). The Moon Pyramid’s Entierro 6 in particular stands out as a singular context with not only the greatest abundance of animal remains (MNI = 75), but also containing the largest concentration of primary burials (MNI = 33) (Fig 1). This represents a unique occurrence of mass animal sacrifice recorded in Mesoamerica during the Classic Period, directly linked to state monumentalism.

**Table 2:** Species distribution of zooarchaeological remains from the Moon Pyramid (Ent. 2, 6, 3 and 5) and Sun Pyramid (PPS OF2).

		Ent. 2			Ent. 6			Ent. 3	Ent. 5			PPS OF2			TOTAL
		MNI	C	I	MNI	C	I	I	MNI	C	I	MNI	C	I	
*Aves*															
*Aquila chrysaetos*	Golden eagle	9	9	-	18	9	9	-	1	1	-	1	1	-	**29**
*Bubo virginianus*	Great horned owl	2	-	2	-	-	-	-	-	-	-	-	-	-	**2**
*Buteo* sp.	Hawk	3	-	3	-	-	-	1	1	-	1	-	-	-	**5**
*B*. *magnirostris*	Roadside hawk	-	-	-	-	-	-	1	1	-	1	-	-	-	**2**
*B*. *jamaicensis*	Redtailed hawk	1	-	1	-	-	-	-	-	-	-	1	-	1	**2**
*Colinus virginianus*	Bobwhite quail	-	-	-	2	-	2	-	-	-	-	-	-	-	**2**
*Columbidae*	Dove/Pigeon	-	-	-	-	-	-	-	3	-	3	-	-	-	**3**
*Columbina inca*	Inca dove	-	-	-	1	-	1	-	-	-	-	-	-	-	**1**
*Corvus corax*	Common raven	2	-	2	-	-	-	-	1	1	-	-	-	-	**3**
*Falco mexicanus*	Praire falcon	1	-	1	-	-	-	-	-	-	-	-	-	-	**1**
UnID Bird		2	-	2	3	-	3	1	-	-	-	-	-	-	**6**
*Mammalia*															
*Ateles geoffroyi*	Spider monkey	-	-	-	-	-	-	-	1	-	1	-	-	-	**1**
*Canis* sp.	Canid	-	-	-	-	-	-	-	1	-	1	-	-	-	**1**
*C*. *lupus baileyi*	Mex grey wolf	1	1	-	9	1	8	17	2	1	1	1		1	**30**
*C*. *latrans*	Coyote	-	-	-	1	-	1	-	-	-	-	-	-	-	**1**
Felidae	Feline	3	-	3	1	1	-	1	3	-	3	-	-	-	**8**
*Panthera onca*	Jaguar	-	-	-	7	1	6	-	2	-	2	-	-	-	**9**
*Puma concolor*	Puma	3	2	1	7	3	4	6	11	1	10	2	-	2	**29**
Leporid	Rabbit/hares	1	-	1	-	-	-	-	-	-	-	-	-	-	**1**
*Lepus* sp.	Hare	2	-	2	-	-		-	1	-	1	-	-	-	**3**
*Sylvilagus* sp.	Cottontail	3	-	3	1	-	1	-	-	-	-	2	-	2	**6**
*S*. *audubonii*	Desert cottontail	1	-	1	3	-	3	-	-	-	-	-	-	-	**4**
*S*. *floridannus*	Eastern cottontail	1	-	1	1	-	1	-	-	-	-	-	-	-	**2**
*Microtus mexicanus*	Mex vole	1	-	1	-	-	-	-	-	-	-	-	-	-	**1**
*Peromyscus* sp.	Deer mouse	-	-	-	-	-	-	-	1		1	-	-	-	**1**
*P*. *maniculatus*	Deer mouse	-	-	-	-	-	-	-	3	-	3	-	-	-	**3**
*Sciurus aureogaster*	Mex gray squirrel	-	-	-	1	-	1	-	-	-	-	-	-	-	**1**
UNID Mammal		1	-	1	2	-	2	-	-	-	-	-	-	-	**3**
*Reptiles*															
*Crotalus* sp.	Rattlesnake	6	6	-	18	18	-	-	9	9	-	-	-	-	**33**
*Anura/Lacertilio*	Frog/lizard	-	-	-	-	-	-	-	1	-	1	-	-	-	**1**
**TOTAL**		*43*	*18*	*25*	*75*	*33*	*42*	*27*	*42*	*13*	*29*	*7*	*1*	*6*	***194***

Ent = Entierro, OF = Ofrenda, MNI = Minimum Number of Individuals, C = Complete, I = Incomplete.

Agents of the Teotihuacan state deliberately selected the most prominent carnivores of the sky (golden eagles), land (pumas, jaguars, and wolves) and liminal zones (rattlesnake) as the core emblems appropriate for state offerings. These beasts were considered to have held diverse sources of power, were revered as master guardian of animals, and were the ultimate symbol of power and rulership throughout Mesoamerica [32, 33]. While there is some degree of diversity among incomplete faunal artifacts, these taxa appear consistently among the complete animals utilized as sacrificial victims across burial contexts. The repeated use of these apex predators in state rituals was mirrored by the rich iconographic repertoire in Teotihuacan mural paintings, which emphasized the same animals [34, 35].

Initial zooarchaeological research on collections from the Moon Pyramid hinted at the presence of an active animal management program within the city confines [36, 37]. Several animals had bone pathologies not usually found in wild carnivores. For example, among the 29 eagles examined, at least eight had a pathology indicating infection, injury, and/or stress that can be related to captive behavior. Three of these individuals exhibit the same pathology on their inner legs (tarsometatasus bone), probably from being tethered. The restrictive device would have led to abrasion and sloughing, that ultimately resulted in the observed bone pathology (Fig 1D). Most likely eagles were kept tethered to perches, similar to the way that modern Native American groups in Southwestern USA still raise eagles for their feathers and for use in sacrificial rites [38]. A number of other pathologies were identified on eagle, puma and wolf skeletons, including broken limbs, bone thinning or osteoporosis on wing elements, abnormal bone fusion (e.g. Fig 1B), and bone modification due to infectious disease.

### Stable Isotopes

**Fig 2:**
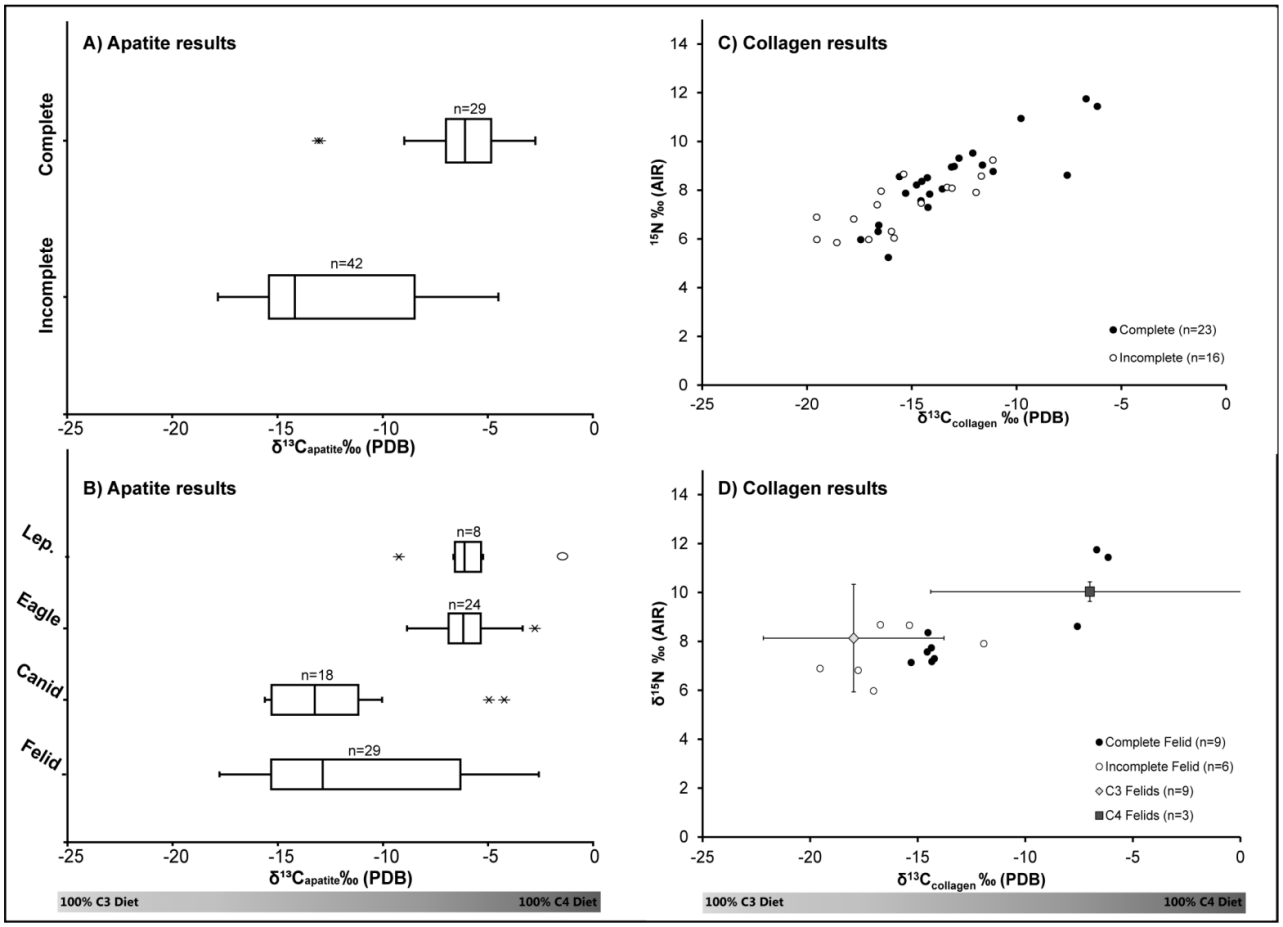
Stable isotope results. δ^13^C_apatite_ values for A) complete and incomplete carnivores (excludes all leporidae) and B) animal type (Lep. = Leporidae). Collagen δ^15^N and δ^13^C_collagen_ values for C) complete and incomplete animal skeletons (excludes leporidae), and D) modern and archaeological felids. Error bars represent two standard divations from average isotopic values of modern felids living in C_3_ (yellow) and C_4_ (red) environments.

Within the Teotihuacan sample (S1 Table) two-tailed t-tests indicated samples from complete skeletons had significantly higher δ^13^C_apatite_ (p<0.001), δ^13^C_collagen_ (p = 0.005), and δ^15^N (p = 0.02) values than did incomplete animals (Table 3, Fig 2A and 2B). The average δ^13^C_apatite_ value for complete individuals (-6.2‰) and secondary animal artifacts (-12.2‰) correlates to approximately 57% and 19% of their diet being composed of C_4_ foods respectively (percent C_4_ calculated following a linear mixing model) [39].

**Table 3:** Descriptive statistics of the δ^13^C_apatite_, δ^13^C_collagen_, and δ^15^N values for complete and incomplete samples (excludes all leporidae, which are rabbits and hares that were found as stomach contents).

	n	Mean	1 σ	df	t Stat	p value
**δ** ^**13**^ **C**_**apatite**_						
Complete	29	-6.2 ‰	2.5‰	69	7.95	*<0*.*001*
Incomplete	42	-12.2‰	3.9‰			
**δ** ^**13**^ **C**_**collagen**_						
Complete	28	-13.3‰	2.9‰	38	2.96	*0*.*005*
Incomplete	18	-15.8‰	2.7‰			
**δ** ^**15**^ **N**						
Complete	28	8.3‰	1.5‰	43	2.41	*0*.*02*
Incomplete	18	7.3‰	1.1‰			

**Fig 3:**
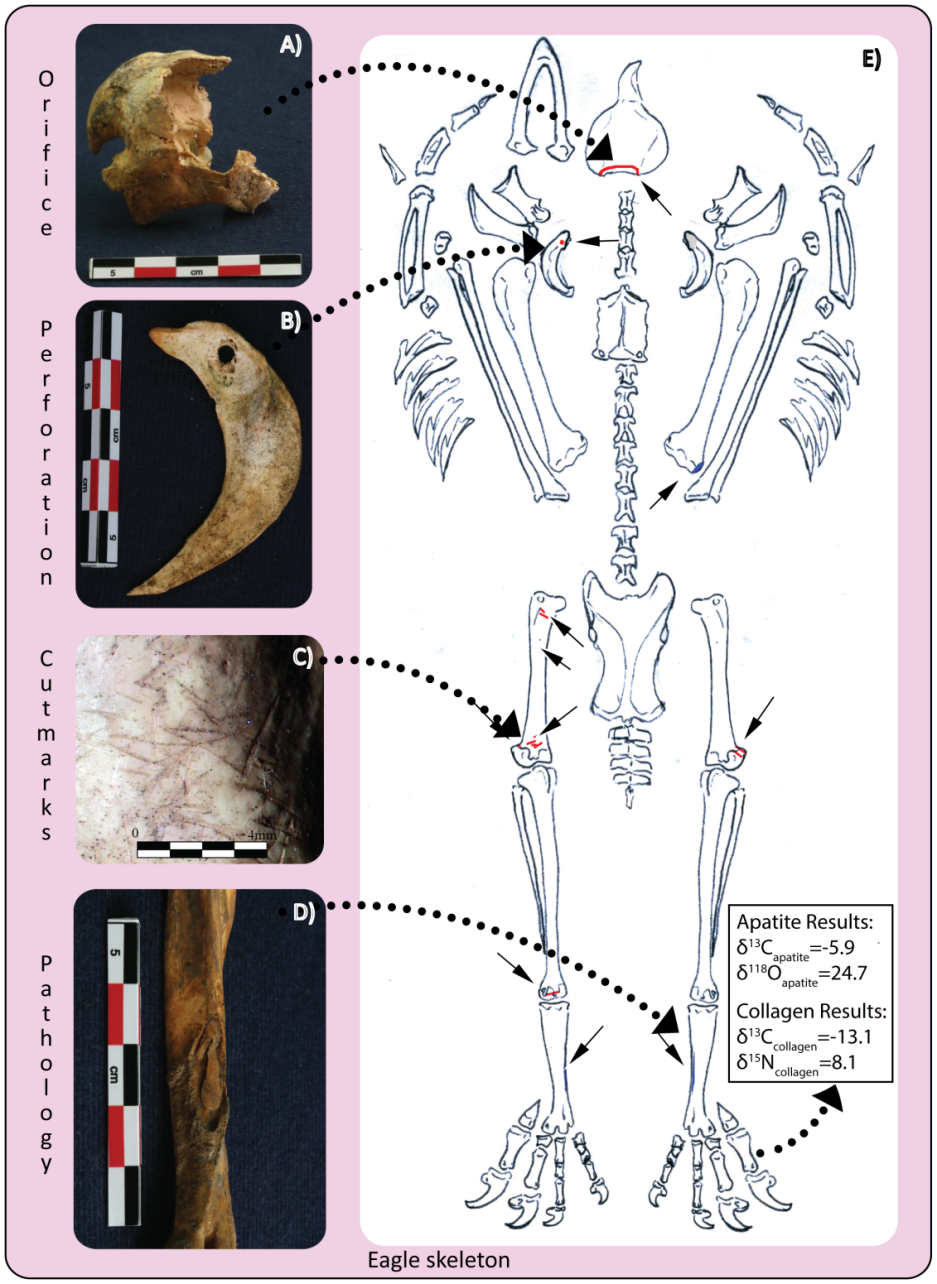
Eagle (Element 2246) placed post-mortem (secondarily) in Entierro 6 with surface modifications. A) Back of the skull with large opening; B) left furculum with perforation; C) cut marks on left femur; D) pathology on inner calf of left tarsometatarsus; E) distribution of cut marks/perforations (red) and pathologies (blue), and isotope results.

There are clear inter-species differences in isotope distributions (Fig 2B). Canids consistently have low to no incorporation of C_4_ based resources, with the exception of two outliers: one complete and one prepared head of a wolf. Eagles, despite representing a mix of sacrificed raptors and prepared skeletons, demonstrate a homogenous diet reliant on C_4_-based foodstuffs. The isotopic signatures concur with the zooarchaeological evidence that eagles with complete skeletal representation can also exhibit extensive post-mortem manipulation of the corpse. For example, Element 2246, an eagle from Entierro 6 has pathologies on both tarsometarsus bones suggestive of captivity, as well as extensive cutmarks and perforations distributed throughout the body indicating it was a secondary burial despite complete skeletal representation (Fig 3). Isotopic data suggest that over half (59%) of its diet was based on C_4_ resources. In some instances, skeletal integrity of the raptor was maintained, probably through taxidermy, placing perforations along the shoulder girdle and extremities. Since the number of eagles for Entierros 6 and 2 were likely predetermined due to its cosmological significance (related to the Mesoamerican calendric cycle), requiring 18 and 9 eagles respectively, eagles were most likely brought into the city confines as chicks in anticipation of their ritual slaughter. However, not all of them were able to be sacrificed alive, thus warranting the use of taxidermy and/or other means of retaining the body intact.

Felid samples had the highest degree of variation in δ^13^C_apatite_ values, and the bimodal distribution indicates that complete (n = 9) and incomplete (n = 20) felids had distinct diets. While collagen was preserved in only 15 of the 29 samples, complete felid burials had significantly (p = 0.03) higher δ^13^C_collagen_ values that averaged -12 ± 3.9‰ (1σ) than did incomplete burials with a mean of -16.4 ± 2.6‰ (1σ) (Fig 2D). When compared to modern wild pumas and other felid species from predominantly C_3_ plant ecosystems (n = 9, puma, bobcat, lynx and tiger) versus felids from C_4_ dominated ecosystems (n = 3, leopard, lion and cheetah) [18, 19, 40], three of the complete archaeological felid isotope values overlapped with the latter range (Fig 2D). Interestingly, these three individuals that most closely mirror isotopic values of felids living in C_4_ based habitats were also animals with extensive pathological evidence for captivity.

So how did these carnivores incorporate C_4_ based resources into their diet during captivity? Two dietary pathways can influence an animal’s δ^13^C isotopic values: direct consumption of maize or indirect consumption by eating C_4_-consuming prey [41, 42]. These two pathways could be distinguished by examining the isotopic signature of the animals found in the stomach contents as well as the nitrogen values of the sacrificed individuals.

Ten of the complete animals were fed leporids (rabbits or hares) prior to their sacrifice. The small bones of those animals were swallowed whole, resulting in fifteen leporid remains in the rib cage of twelve carnivores. In some instances several fragments were burnt, directly attesting to the practice of artificial feeding. Stable isotope analyses on these leporids indicate that they were reliant on C_4_ plants, with δ^13^C_apatite_ values averaging -5.9 ± 2.1‰ (1σ), with a range of -9.3‰ to -1.6‰ corresponding to diets containing approximately 38% to 86% C_4_ foods.

Most fed carnivores were provided leporids before their sacrifice (this excludes rattlesnakes, which usually ate rodents), and the leporids’ higher δ^13^C_apatite_ values suggest they were raised in captivity. Archaeological data from the Oztoyahualco apartment compound at Teotihuacan suggest rabbits were raised for human consumption [43]. These lagomorphs would have supplied a constant and predictable source of meat protein to raise these specialized carnivores.

Experimental and field studies indicate that mammals typically exhibit δ^15^N values 3–4‰ higher than their primary protein source [19, 44]. Similar patterns are observed between carnivores and leporids within the analyzed samples. Leporid δ^15^N values average 5.2 ± 1.3‰ (1σ), while carnivore averages range between 7.4 ± 1.9‰ (1σ) (canids) to 8.1 ± 1.6‰ (1σ) (felids). Felids, however, demonstrate extensive δ^15^N variation. Two pumas in particular exhibit exceptionally high δ^15^N (11.8‰ and 11.4‰) and δ^13^C_collagen_ (-6.7‰ and -6.1‰) ratios in comparison to all the other complete felid skeletons (Fig 2D). Thus these two pumas must have been eating meat readily available in an urban metropolis composed of not only maize-eating herbivores but also other omnivores that were heavily reliant on C_4_ foods.

**Fig 4:**
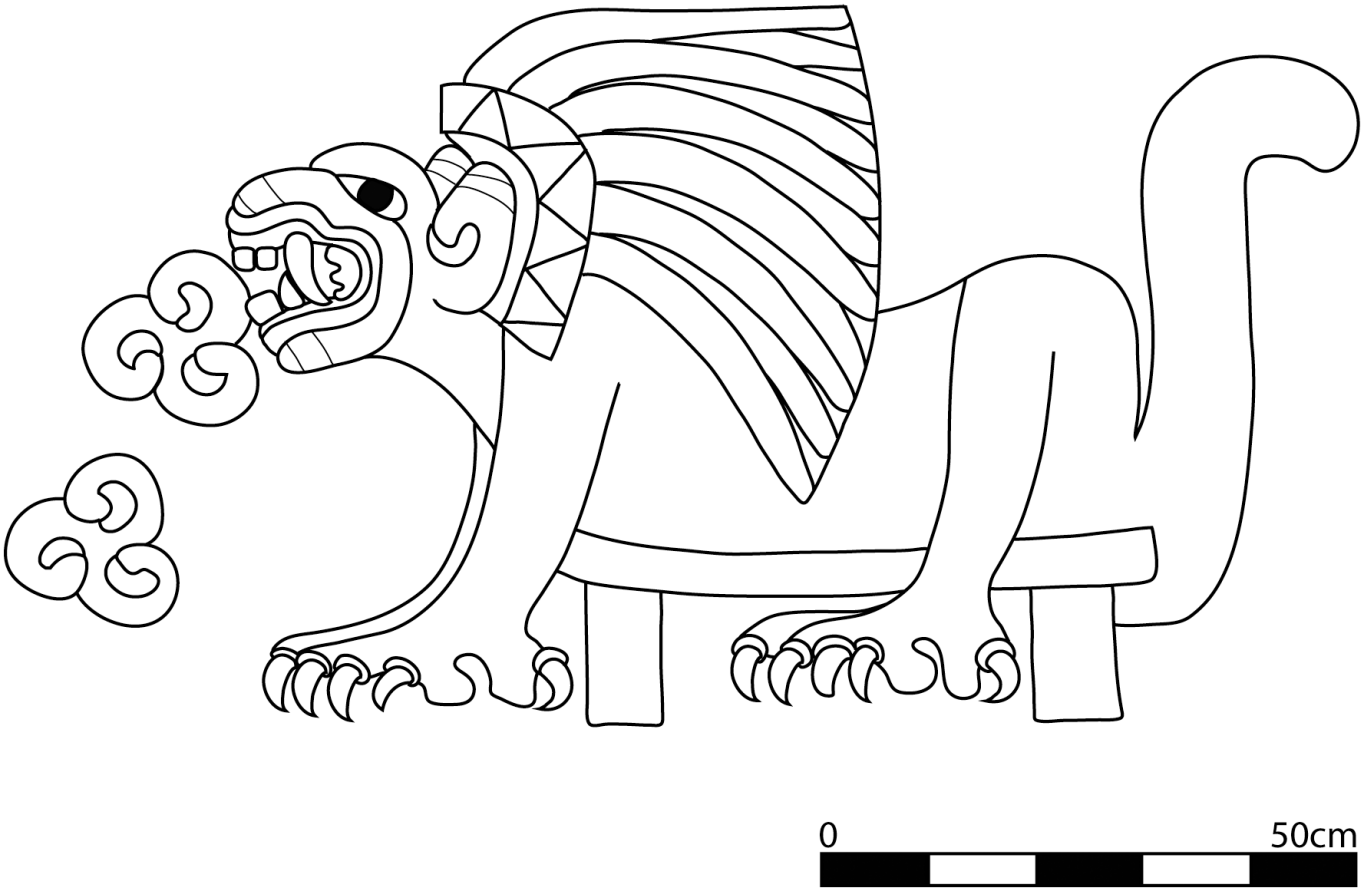
Line drawing of puma devouring hearts. From the Tetitla apartment compound, Portico 13, Mural 3. Drawing by N. Sugiyama.

Two possible protein sources that fit these criteria are humans and dogs. The consumption of humans by captive felines is plausible given the descriptions by Spanish conquistadors of how ferocious carnivores housed in Moctezuma’s zoo were fed the remains of humans used in sacrificial rites, including the bodies of conquistadors obtained in battle [1]. In fact there is a long tradition associating wild carnivores to human sacrifice throughout Mesoamerica. At the post-Teotihuacan sites of Tula and Chichén Itzá, felids are depicted devouring human hearts on stone and stucco friezes in the ceremonial center. Ample iconographic evidence at Teotihuacan similarly depicts the active role of carnivores in human sacrificial rites. Carnivores are illustrated in procession holding large sacrificial knives, in military regalia and even devouring human hearts [34] (Fig 4). The felids from the ritual deposits at Teotihuacan that were interred alongside beheaded human sacrificial victims may have been actively involved in state-ritualized activities beyond their roles as sacrificial victims.

Unfortunately it is difficult to distinguish between humans and dogs based on isotopic ratios. Dogs have long established roots in Mesoamerican domestic life, being cohabitants of human settlements and eating trash including human feces, so much so that they have been used as proxies for human dietary regimes in areas where isotopic studies on human skeletal remains are not feasible [45]. As one of the major domesticated animals in Mesoamerica that was also utilized as a staple protein source, the dog must be considered as a potential food source for the pumas with elevated nitrogen and carbon isotope ratios. Most likely, these two felids were fed a mixed diet of maize-raised lagomorphs supplemented with dog and/or human meat. It is important to emphasize that as bone collagen values reflect an average total diet at the rate in which bone remodels (in this case reflecting the young-adult puma’s lifetime average), this value does not reflect isolated feeding events. There is one wolf skeleton with a somewhat elevated δ^15^N value (10.9‰) from the same dedicatory cache, suggesting a similar scenario.

## Conclusion

Zooarchaeological and isotopic evidence from Teotihuacan demonstrate the carnivores utilized in state rituals at Teotihuacan were a heterogeneous group: some of the sacrificed individuals were kept in captivity, others were brought in from the wilderness, and some were used to produce ritual paraphernalia. Canid and felid skeletons demonstrate that sacrificial victims were often kept in captivity while faunal products used as ritual paraphernalia originated from a wild population. In contrast, both sacrificed and prepared skeletons of eagles demonstrate some degree of captive management. Compared to the well-established breeding programs recorded at the Aztec capital, the animals found at Teotihuacan probably reflect initial experimentation in manipulating dangerous and specialized carnivores. This process probably involved capturing the animals alive while they were young to be raised within the city confines on small animals, most likely rabbits and hares that ate an exclusively C_4_ based diet. In some instances, rabbits and hares may have been supplemented by other omnivores, including dogs and possibly even humans.

Specimens like the eagle, Element 2246, attest to the hardships in learning to manipulate highly specialized carnivores. Keeping a captive population alive must have been difficult, sometimes resulting in accidental deaths of their captive eagles that were then expediently prepared to keep the body intact; these stuffed cadavers would have participated alongside live eagles as if they were also “sacrificed”. Caring for and manipulating the region’s most dangerous apex predators sometimes required the use of brute force as evidenced by an unnaturally high frequency of healed fractures, violent injuries, bone deformity and disease.

The broader implications of these finds are apparent; they not only provide the earliest direct evidence for ritualistic management of carnivores but also demonstrate a fundamental shift in past human-carnivore interactions that was intrinsically tied to the development of one of the most empowered ceremonial landscapes in Mesoamerica. As both the jaguar and Mexican grey wolf are currently on the endangered species lists, the archaeological and isotopic data presented provide a window into understanding human-animal encounters of these endangered species through the *long dureé*, beginning with the earliest documentation of how these animals were captured and managed. These data also speak to a direct connection between carnivore manipulation and state power. There was an impetus to bring live carnivores into the city as cubs/chicks to be raised within the urban metropolis as sacrificial victims *par excellence*. It is not a coincidence that such active animal management programs coincided with the development of large monumental structures where animals were embedded into pyramids as key symbols of the Teotihuacan state.

## Supporting Information

S1 Table Raw data of isotope analysis before data corrections. * Samples that were dropped based on diagenesis tests. Comp? = Complete individual?, Col. Weight = collagen weight, % yield = % collagen yield, Ent. = Entierro, OF = Ofrenda (Sun Pyramid).
(DOCX)

S2 Table Carbonate and collagen samples used and dropped based on diagenesis tests. Ent. = Entierro, OF = Ofrenda.
(DOCX)

S1 Text Diagenesis. (DOCX)
