# Supplementary material for: Stable Isotopes and Zooarchaeology at Teotihuacan, Mexico Reveal Earliest Evidence of Wild Carnivore Management in Mesoamerica
Source: PLoS One. 2015 Sep 2;10(9):e0135635. doi: 10.1371/journal.pone.0135635 (PMC4557940; doi:10.1371/journal.pone.0135635)
Supplement: S1 Table — * Samples that were dropped based on diagenesis tests. Comp? = Complete individual?, Col. Weight = collagen weight, % yield = % collagen yield, Ent. = Entierro, OF = Ofrenda (Sun Pyramid). (DOCX) [file pone.0135635.s001.docx]

**S1 Table. Raw data of isotope analysis before data corrections.** * Samples that were dropped based on diagenesis tests. Comp?= Complete individual?, % yield=% collagen yield, Ent.=Entierro, OF=Ofrenda (Sun Pyramid)****
