# Supplementary material for: Stable Isotopes and Zooarchaeology at Teotihuacan, Mexico Reveal Earliest Evidence of Wild Carnivore Management in Mesoamerica
Source: PLoS One. 2015 Sep 2;10(9):e0135635. doi: 10.1371/journal.pone.0135635 (PMC4557940; doi:10.1371/journal.pone.0135635)
Supplement: S2 Table — Ent. = Entierro, OF = Ofrenda. (DOCX) [file pone.0135635.s002.docx]

**S2 Table**. **Carbonate and collagen samples used and dropped based on diagenesis tests**. Ent.=Entierro, OF=Ofrenda.
