# Supplementary material for: Stable Isotopes and Zooarchaeology at Teotihuacan, Mexico Reveal Earliest Evidence of Wild Carnivore Management in Mesoamerica
Source: PLoS One. 2015 Sep 2;10(9):e0135635. doi: 10.1371/journal.pone.0135635 (PMC4557940; doi:10.1371/journal.pone.0135635)
Supplement: S1 Text — (DOCX) [file pone.0135635.s003.docx]

**S2 Text: Diagenesis**

Preservation and excavation/consolidation procedures varied significantly for each of the dedicatory caches. In some cases, the bone had completely deteriorated, warranting the immediate application of consolidants to move the skeletal elements. Thus determining the effects of diagenesis was a major concern for the project. As physical and biological processes of the burial environment differentially affect inorganic and organic components of bone, distinct methods were applied to determine diagenesis for apatite and collagen samples (1).

Degree of diagenesis of bone carbonate samples were assessed utilizing the Fourier-Tranform Infrared Spectrometer with attenuated total reflectance (ATR) attachment at the UCSD Department of Chemistry and Biochemistry Laboratory. Two measures were utilized in this analysis: C/P ratios and Infrared Splitting Factor (IR-SF)(2-4). IR-SF values, calculated as [565_ht_+605_ht_]/590_ht_, where X_ht_ is height at X wavenumbers (5), above 4.0 usually indicate extremely degraded or burnt bone (FTIR-KBr values) (2, 4). Compared to the FTIR-KBr methods, the FTIR-ATR values tend to average 0.5 higher in IR-SF splitting factor while there is not much difference in C/P ratio (6). Since none of the values in the present study were above 4, this distinction did not affect sample selection and none of the samples were discarded based on this criteria. C/P ratios (1415_ht_/1035_ht_) on modern bone averages approximately 0.5 and values below 0.1 signify the presence of extensively degraded minerals (2, 4). A total of 37 out of 87 bone apatite samples with values below 0.1 (not rounding up) were not utilized in the present study (S2 Table). No diagenesis tests were conducted on tooth samples as enamel is not as susceptible to diagenetic processes as bone. In cases where bone preservation of an entire burial did not meet requirements set by diagenesis tests, like bones from Entierros 3 and 5, such tooth samples sometimes provided the only means of obtaining any isotope values.

Collagen integrity was evaluated by calculating the % collagen yield and C/N ratios. Following pertinent literature, a cut off of one percent collagen yield was set (7, 8). As modern bone C/N ratios, calculated as (%C/12)/(%N/14), range between 2.9 and 3.6 (1, 8, 9), any sample that did not fall within this range were also excluded from the study. Together, 24 out of 75 collagen samples were excluded.

Diagenesis tests indicated that taphonomical processes from Entierro 3 and 5 greatly affected bone preservation, making both carbonate and collagen isotope results unreliable. Only teeth samples provided the bulk of the carbonate samples from these contexts. Unfortunately, this meant that no collagen results were obtained from Entierro 5 and only one bone from Entierro 3 could be included in this study. Nonetheless, the total sample size still remains large with 105 carbonate and 51 collagen samples spanning various species and contexts, making this a representative sample for the present study.

**References**

1. Schoeninger MJ, Moore KM, Murray ML, & Kingston JD (1989) Detection of Bone Preservation in Archaeological and Fossil Samples. *Applied Geochemistry* 4(3):281-292.

2. Wright LE & Schwarcz HP (1996) Infrared and Isotopic Evidence for Diagenesis of Bone Apatite at Dos Pilas, Guatemala: Palaeodietary Implications. *J Archaeol Sci* 23(6):933-944.

3. Weiner S & Bar-Yosef O (1990) States of Preservation of Bones from Prehistoric Sites in the Near East: A Survey. *J Archaeol Sci* 17(2):187-196.

4. Smith CI, Nielsen-Marsh CM, Jans MME, & Collins MJ (2007) Bone Diagenesis in the European Holocene I: Patterns and Mechanisms. *J Archaeol Sci* 34(9):1485-1493.

5. Garvie-Lok SJ, Varney TL, & Katzenberg MA (2004) Preparation of Bone Carbonate for Stable Isotope Analysis: The Effects of Treatment Time and Acid Concentration. *J Archaeol Sci* 31(6):763-776.

6. Beasley MM, Bartelink EJ, Taylor L, & Miller RM (2014) Comparison of transmission FTIR, ATR, and DRIFT Spectra: Implications for Assessment of Bone Bioapatite Diagenesis. *J Archaeol Sci* 46(0):16-22.

7. Schwarcz HP & Schoeninger MJ (2011) Stable Isotopes of Carbon and Nitrogen as Tracers for Paleo-Diet Reconstruction. *Handbook of Environmental Isotope Geochemistry*, ed Baskaran M (Springer-Verlag, Berlin, Heidelberg), Vol II, pp 725-742.

8. Ambrose SH (1990) Preparation and Characterization of Bone and Tooth Collagen for Isotopic Analysis. *J Archaeol Sci* 17(4):431-451.

9. DeNiro MJ & Weiner S (1988) Chemical, Enzymatic and Spectroscopic Characterization of “Collagen” and Other Organic Fractions from Prehistoric Bones. *Geochim Cosmochim Acta* 52(9):2197-2206.

10. Warinner C & Tuross N (2009) Alkaline Cooking and Stable Isotope Tissue-Diet Spacing in Swine: Archaeological Implications. *J Archaeol Sci* 36:1690-1697.
